# A Single Long Day Triggers Follicle Growth in Captive Female Great Tits (*Parus major*) in Winter but Does Not Affect Laying Dates in the Wild in Spring

**DOI:** 10.1371/journal.pone.0035617

**Published:** 2012-04-24

**Authors:** Luc te Marvelde, Sonja V. Schaper, Marcel E. Visser

**Affiliations:** Department of Animal Ecology, Netherlands Institute of Ecology (NIOO-KNAW), Wageningen, The Netherlands; Pennsylvania State University, United States of America

## Abstract

In many forest passerine bird species, rapid climate warming has led to a phenological mismatch between the period of maximum nestlings' food requirements and the period of maximum food availability (seasonal caterpillar biomass peak) due to an insufficient advancement of the birds' laying dates. The initiation of laying is preceded by the development of the gonads, which in birds are regressed outside the breeding season. Increasing day length in late winter and early spring triggers a cascade of hormones which induces gonadal development. Since day length is not altered by climate change, one potential restriction to advancing laying date is the seasonal timing of gonadal development. To assess the importance of gonadal growth for timing of reproduction we experimentally manipulated the timing of gonadal development. We show that the growth of the largest follicle of captive female great tits (*Parus major*) increased after being exposed to just a single long day in winter (20 hours of light followed by 4 hours darkness). We then photostimulated wild female great tits from two study areas in a field experiment in spring for a single day and determined their laying date. These populations differed in the availability of food allowing us to test if food availability in combination with photostimulation affected egg laying dates. Despite an expected difference in the onset of gonadal growth, laying dates of photostimulated females did not differ from control females in both populations. These results suggest that wild great tits are not restricted in the advancement of their laying date by limited gonadal development.

## Introduction

Breeding success largely depends on the timing of breeding relative to the timing of maximum food availability [1]–[3]. Many forest passerine bird species in temperate regions feed their young with caterpillars, which occur only in a short period of time during spring (caterpillar biomass peak). Due to increased spring temperatures, the window in which food availability is high has shifted forward in time over the last 25 years [4]. As a result, the optimal timing for breeding advanced, but many species, like the great tit, have not adjusted their timing sufficiently, causing them to breed too late [4]–[6].

The reproductive system of most seasonally breeding birds, including great tits, shows a clear seasonal pattern [7], [8]. Gonads are regressed during winter, grow slowly during late winter and grow rapidly in spring until fully developed. Gonads are regressed again after the breeding season. The rapid growth in spring is affected by increasing day length, which causes the release of gonadotrophins [7], [9], [10]. Although it has been shown that temperature can affect the speed at which gonads develop in great tits breeding in Southern latitudes [11], the speed of gonadal development in great tits breeding in more Northern latitudes is not accelerated by increasing spring temperatures (The Netherlands [12], Scandinavia [11]). In these latitudes, gonadal growth is driven by photoperiod [11]. Increasing spring temperatures due to global warming will therefore not advance the birds' readiness to reproduce. As climate change does not affect the seasonal change in photoperiod, a possible reason as to why great tits are not advancing their laying date adequately is that gonads are not fully developed early enough to allow early egg laying.

To test whether gonadal development is hampering early egg laying, gonadal development needs to be experimentally advanced. This could be done by manipulating the photoperiod a bird experiences, as is shown in an experiment where blue tits (*Cyanistes caeruleus*) in captivity (with *ad libitum* food) could be tricked into laying their eggs in winter (January) by exposing them to long days from December onwards [13]. Under a natural photoperiod, egg laying in January is not possible as the reproductive system will not be fully developed at that time.

Although the experienced photoperiod of captive birds is easily manipulated, photostimulating birds in the field has many practical problems, such as fitting a large number of nest boxes with a light, batteries and a timer. More importantly, birds do not always sleep in a particular nest box in the period before egg laying and thus it is difficult to determine which bird is photostimulated and by how much. Taking wild birds into captivity for photostimulation treatment for long periods can cause problems as breeding vacancies as a result of the removal of territorial birds are filled within a few days by unpaired birds, which may lead to fights after release of the original territory holder or its female (pers. comm. P. de Goede (NIOO-KNAW)).

Previous experiments have shown that the exposure to ‘a single long day’ can affect levels of hormones involved in reproduction. Nicholls and colleagues [14] kept Japanese quail under 8L∶16D and gave them a single long day of 20L∶4D resulting in an increase of luteinizing hormone (LH) and follicle stimulating hormone (FSH) within four hours of the end of the long day. These birds were kept in constant darkness thereafter and their LH and FSH levels decreased slowly over the next 8 to 10 days. Creighton and Follett [15], who performed a similar experiment, kept Japanese quails under short day lengths after just one long day and report that LH remained elevated for three days after photostimulation. Follet and colleagues [16] have shown that LH levels of white-crowned sparrows (*Zonotrichia leucophrys gambelii*) transferred from short (8L∶16D) to long days (20L∶4D) increased six-fold in five days, with the largest increase after the first day (three-fold increase). Saab and colleagues [17] has shown that a single long day increased gonadotrophin-releasing hormone and LH concentrations in white-throated sparrows (*Zonotrichia albicollis*). A single long day also affects gonadal growth of both male and female Song sparrows (*Melospiza melodia*). Wingfield [18] showed that after one single long day, females' gonads grew for up to 60 days, even though the changes in LH and FSH were only present for a few days. In all of the experiments above food was available *ad libitum*. If a single long day would affect gonadal growth in wild female great tits, it would allow us to test the hypothesis that laying dates are restricted by photo-induced gonadal growth.

Outside the breeding season gonads are regressed, implying that there are costs connected with having and/or maintaining fully developed gonads. These costs can be present in terms of increased risk of predation due to lower aerial maneuverability and take-off ability [19]–[21], but might also involve energetic maintenance costs. Thus, it is likely that advancing gonadal growth in spring also comes with a cost. If energetic costs restrict early gonadal development, only birds in habitats with high food availability might be growing their gonads as a reaction to a single long day.

The aim of this study was to determine (i) if a single long day induces gonadal growth in captive female great tits and (ii) if a single long day in spring affects laying dates in two field populations which differ in the availability of supplementary food in the period before and during egg laying. If gonadal growth restricts early egg laying, we expect photostimulated birds to lay earlier compared to control birds in both study areas. If gonadal growth is restricted by a combined effect of photoperiod and food availability, we expect only those birds in the population with available supplementary food during the pre-laying period to advance egg laying.

## Results

### Aviary experiment

**Figure 1:**
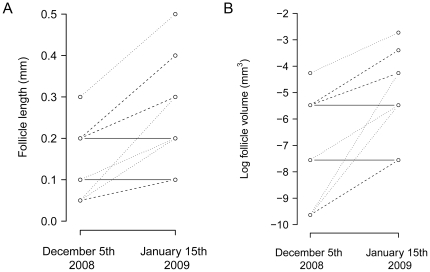
Effect of photostimulation on follicle growth in captive great tits in winter. Follicle length (*A*) and volume (*B*) of the three experimental groups in the aviary experiment before and one month after the photostimulation. All birds were kept in an outdoor aviary and only moved indoor for the photostimulation treatment. Of the females of which we successfully measured the largest follicle in December and January, two birds were kept under natural light conditions (solid lines), 3 individuals were given one long day (dashed lines) and 4 individuals were given two long days (seven days apart; dotted lines).

Gonadal growth of female great tits in captivity could only be determined for 9 out of the 15 females due to technical difficulties (two control females, three females photostimulated once and four individuals photostimulated twice in December were measured). Females that were photostimulated once or twice (20L∶4D) showed gonadal growth one month after the treatment (One-sample Wilcoxon signed rank test: V = 28, *P* = 0.016), whereas both females of the control group did not show gonadal growth. Gonadal growth of females which were photostimulated once did not differ from females that were photostimulated twice (Two-sample Wilcoxon signed rank test: W = 5, *P* = 0.86; see Fig. 1).

### Field experiment

**Figure 2:**
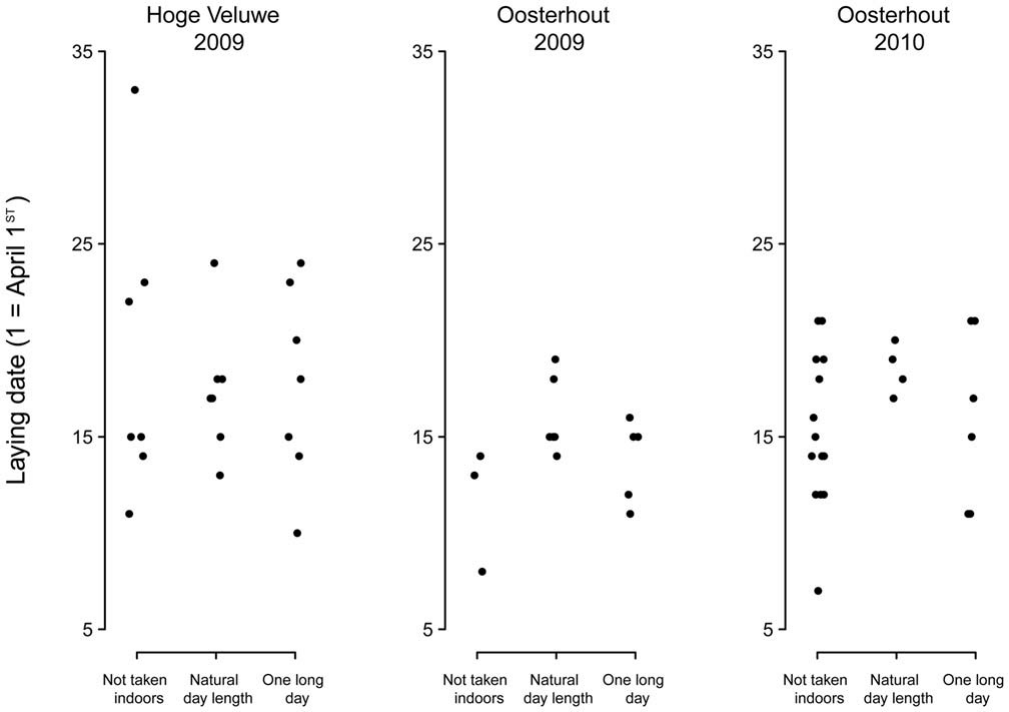
Effect of photostimulation on laying dates in free living great tits. One third of the females encountered during a night check of all nest boxes were not taken indoors, 1/3 were taken into captivity and kept under natural light regime and 1/3 were taken into captivity and kept under a long light regime (according to figure 3). Note that random jitter is applied (on the X-axis only) to separate overlapping data point.

In total, 63 out of 103 females in the field experiment started egg laying in our study area (Table 1). Females started laying on average (± SE) on April 18.0 (±1.17), 14.3 (±0.69) and 16.7 (±1.0) in the Hoge Veluwe 2009, Oosterhout 2009 and 2010, respectively. Laying dates of first eggs did not differ between photostimulated and control female great tits in either of the two years and in either of the two study areas (P>0.22 for all comparisons; Table 2; Fig. 2).

**Table 1:** Sample sizes of the field experiment to study the effect of photostimulation on laying dates in wild living great tits (*Parus major*).

		*Treatment*	*Sample size*	*Birds with laying date*	*Percentage with lay date*
Oosterhout	2009	Indoor – one long day	10	6	59%
		Indoor – natural day length	10	6	
		Not taken indoors	7	4	
	2010	Indoor – one long day	10	7	72%
		Indoor – natural day length	10	5	
		Not taken indoors	16	14	
Hoge Veluwe	2009	Indoor – one long day	15	7	53%
		Indoor – natural day length	14	7	
		Not taken indoors	11	7	
Total indoor			69	38	55%
Total not taken indoor			34	25	73%
Totals			103	63	

We created 3 experimental groups: (i) photostimulated females (ii) females which experienced natural day length in captivity and (iii) females which experienced natural day length in the field. Females from group (i) and (ii) were kept indoors for one day and two nights after which they were released in the field at the location where they were caught.

**Table 2:** Results of the statistical analyses (ANOVAs) of the effect of photostimulation on first egg laying dates (date at which the first egg of a clutch is laid) of wild great tits (*Parus major*) in the field experiment.

*Subset*	*Variable*	*df*	*Error df*	*Sum of squares*	*Mean squares*	*F*	*P*
Oosterhout	Treatment * year	2	33	1.05	0.53	0.05	0.96
Oosterhout	Treatment	2	36	38.39	19.19	1.59	0.22
2009	Treatment * location	2	30	40.35	20.18	0.96	0.39
2009	Treatment	2	33	3.02	1.51	0.06	0.94

The experiment had three treatments: indoor photostimulated; indoor natural light regime; not taken indoors. The experiment was done in two locations in 2009 and in one location in 2010. We divided the analyses in two; i) only data from the Oosterhout population (2009 and 2010) and ii) only data from 2009, where the experiment was done in Oosterhout and Hoge Veluwe population.

## Discussion

This study aimed to test the hypothesis that timing of egg laying is restricted by the timing of gonadal development, which is under photoperiodic control. We showed that, even in winter (average daily temperature in the outdoor aviary in December 2008 = 1.8°C, January 2009 = 0.1°C; February = 2.7°C), gonadal growth is initiated after exposing captive female great tits to ‘a single long day’ (20L∶4D). In a field experiment in spring, however, free living female great tits which were given a single long day did not advance egg laying, either in the study area with or without good food conditions and the availability of supplementary food in the period before egg laying. Although we did not measure gonadal development prior to egg laying in the field study and can therefore not confirm that our ‘single long day’ treatment also worked in spring, these results suggest that the seasonal timing of gonadal growth does not play a major role in restricting great tits from advancing their laying date.

Follicles of captive female great tits which were exposed to a single long day in winter started growing. As gonads are in a regressed state outside the breeding season and only start growing very slowly in early winter, follicles were still small in December (maximum 0.3 mm long). It is therefore difficult to measure their size: in 3 out of 15 cases, the follicle size in December was too small to be measured precisely. Follicle sizes in January ranged from 0.1 mm to 0.5 mm, which is still small compared to the accuracy with which we can measure them (0.1 mm). Although measuring follicles in winter is difficult, we have confidence in our measurements.

Both populations have a long history of selection for early laying: reproductive success, measured as the number of fledged offspring that recruited in the breeding population in the next year, was higher for early breeding females for 21 out of 25 years in Oosterhout, and for 23 out of 25 years in the Hoge Veluwe [4]. If gonadal growth would restrict egg laying, this would most likely occur in years with high spring temperatures since warm springs lead to early egg laying. Temperature in the period 16 March until 20 April correlates well with laying dates [4]. In 2010, mean temperature in this period was high and indeed, the first pair that started egg laying in the Oosterhout study population was the earliest recorded laying date in the last 55 years in this study area. Although laying dates are early in years with warm springs, gonadal growth is not affected by temperature in the period before egg laying [12]. Therefore we would expect an effect of photostimulation (especially in 2010) if gonadal growth was restricting egg laying dates.

Although follicle volumes increased after one day of photostimulation in captivity during winter, photostimulation in spring did not affect laying dates in the field. There are a number of potential explanations.

We did not measure gonadal growth in a subset of the photostimulated animals in March. It is therefore possible that the photostimulation in March did not result in gonadal growth, causing the lack of effect in laying dates. To our knowledge, no studies have focused on the seasonal variation in the strength of the response in hormonal change or gonadal growth to a single long day. However, Silverin [8] caught male great tits during different months of the year and exposed (a part of) them to a 20L∶4D light regime for 100 days. Testis growth of male great tits caught in December was less than one millimeter after 10 days of photostimulation, whereas male great tits caught in March grew their tested on average just over 2 mm. Thus, at both dates photostimulation leads to a reaction that was adequate for that time and developmental stage and it is therefore likely that follicle growth of the female great tits in our field experiment was stimulated by our experimental treatment in March.

Since the natural day length is shorter in winter than in spring, the photostimulation in winter was a relatively stronger stimulation compared to the photostimulation in spring. Silverin [8] measured testes growth of male great tits exposed to two different light regimes (14L∶10D and 20L∶4D) and showed that the gonadal maturation was faster in he 20L∶4D group compared to the 14L∶10D group. In our experiment in spring, the increase in day length was still 9 hours and 15 minutes for the photostimulated group, which is more than the increase in day length from 8L∶16D to 14L∶10D in the experiment from Silverin [8] which resulted in clear effects on hormones and gonadal development. We therefore believe the photostimulation treatment in spring is strong enough to evoke a response in gonadal growth.

Another possible explanation why photostimulated females did not advance egg laying is that, besides the primary predictive cue of photoperiod, supplementary cues are used to time egg laying [22], for example the increase in spring temperature [12], [23]. One of the supplementary cues might be food availability. During the aviary experiment, as well as in most other experimental studies [12], [23], food and water were given *ad libitum* while the females in the field encountered all kinds of stressors (predation risk, lower food availability, inter-species interactions et cetera). In addition, eggs are laid in cold weather conditions under which foraging efficiency is low [24] and energetic costs are high [25], [26]. Therefore, adverse food conditions can restrict growth of gonads (either acting as a cue or as an energetic constraint). Perfito *et al* [27] showed that male zebra finches (*Taeniopygia guttata guttata*) under long photoperiod but with food restrictions did not develop their testis, similar to those under short day lengths, while birds under long photoperiod with *ad libitum* food did. Zebra finches, however, are opportunistic breeders that have evolved to use food availability as a cue, since they live in areas where food availability is unpredictable and do not follow a seasonal pattern [28]. Testis size of male European starlings kept in aviaries was not affected by a food restriction [29], however, as the birds were able to maintain body weight during the treatment they were possibly not restricted enough. Also, in most physiological studies like these, males are used, while it is the females that determine the timing of reproduction [30]. It is likely that the female great tits in our field experiment were food restricted. If this would be the case, we would expect females in Oosterhout (which had a richer food supply) to advance egg laying compared to the control females, but not the Hoge Veluwe females. However, photostimulated females from the Oosterhout population also did not advance laying compared to the control groups in neither of two years. Therefore, we hypothesize that other supplementary cues, like temperature, or perhaps the availability of insects, prevented photostimulated females from laying early relative to the phenology of their environment.

While egg laying can not start before males *and* females have fully developed reproductive organs, we only photostimulated female great tits in the field experiment. Field observations showed that the male reproductive system is functioning well before that of the females [22]. Gonadal measurements of great tit breeding pairs in captivity confirmed this by showing that males have mature gonads sometimes weeks earlier compared to the exponential growth phase of female follicles [23], [31]. Therefore, laying dates in wild birds are not likely to be restricted by the development of the male reproductive system.

It is important to know which factors hamper the lack of shift in laying dates because these can have different implications on how to adapt to future climate change. If a shift in laying date is hampered by gonadal development (which our result suggest is it not), birds have to adjust their rules in which day length is used as a cue. Since climate change is not affecting day lengths, gonadal development is likely to restrict a shift in laying dates in the future. At the moment there seems be other reasons why the shift in the advancement of laying date lags behind this shift in the phenology of the food, leading to an increasing phenological mismatch between food availability and food requirements over the last decades. Future temperature increase will further this mismatch. A better understanding of the causes and consequences of the (in)ability of birds to adapt their timing of reproduction to restore the synchrony with their prey is important and will provide insights into the effects of future climate change on population viability [32].

## Methods

### Ethics Statement

The experiments reported here comply with the current law in The Netherlands and were carried out under licenses of the Animal Ethics Committee of the KNAW (Royal Netherlands Academy of Arts and Sciences, protocol CTE.08.10 & CTE 09.01).

*Study areas –* This study was carried out in two study areas, Hoge Veluwe and Oosterhout (the Netherlands), about 50 km apart. Strong natural selection for early laying females exists in both populations [6]. The study sites were chosen for this experiment because food availability in the period before egg laying differs between them. National Park ‘De Hoge Veluwe’ (52° 02′ 07″ N 5° 51′ 32″ E) is a mixed forest on poor sandy soils, while Oosterhout (51° 52′ 22″ N 5° 50′ 22″ E) is a rich deciduous forest on rich river clay. Besides finding food in the rich undergrowth, females of the Oosterhout population regularly fly to the nearby village (maximum distance 1 km) to feed on the abundant supplemented food (observed during radio tracking, unpublished data LtM & MEV). This food was available until most birds laid their eggs and consisted mainly of fat and peanuts. Over the last 25 years, natural selection favored early breeding in 21 of the past 25 years in Oosterhout and in 23 out of 25 years in the Hoge Veluwe [4].

### Aviary experiment

To test if one (or two) long days initiate gonadal growth, we caught 15 wild female great tits at the end of November 2008 around the Netherlands Institute of Ecology, Heteren (The Netherlands; 51°57′20″N–5°44′34″E). All females were housed in one large outdoor aviary under natural light and temperature conditions and *ad libitum* food and water. Six days before the light treatment (December 5^th^ 2008), length of the largest ovarian follicle of all females was measured during laparotomy without knowledge of the treatment each female would be assigned to. To measure follicle development, a small incision was made between the last two ribs on the left side. By parting the ribs slightly, length of the largest follicle was measured to the nearest 0.1 mm with an ocular scale. When the length of the largest follicle was too small to be measured (significantly smaller than 0.1 mm) we reported a length of 0.05 mm (*n* = 3 in December). All laparotomies were carried out by SVS under light Isoflurane anesthesia. Follicle volume was calculated as V = 4/3 · π · a^3^ where *a* is ½ the length of the follicle.

**Figure 3:**
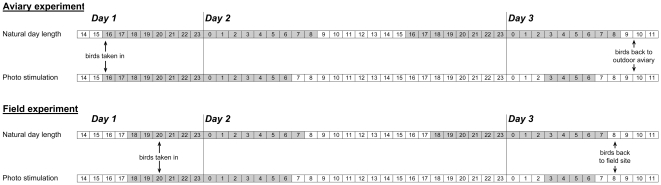
Schematic overview of the photostimulation treatment for both the aviary and the field experiment. For the experiment in captivity (December 2008), females were moved from the outdoor aviary to our indoor facilities after sunset, where a part of the females were kept under natural day length and a part were kept in a different room under a long day length (20L∶4D). Females were moved to their outdoor aviary in the morning of day three. For the field experiment (March 2009 and 2010), females were brought from the field into out indoor facilities for a similar treatment. On the morning of day three, all females were released in the field at the location where they were caught. Grey bars resemble darkness while open bars represent light periods; numbers in the bar are the hours of the day.

For the first photostimulation treatment (December 11^th^ 2008), all females were moved indoors after sunset into individual cages in two separate rooms (see Fig. 3 for a schematic overview of the treatments). Ten females in one room were exposed to light for 20 hours (7AM–3AM), then dark for four hours, after which the lights were turned on again at 7AM the following day. Five control females in the second room were kept under the natural light regime (light from 8:40AM to 4:30PM). All females were returned to the outdoor aviary the day after the photostimulation treatment. Seven days later (December 18^th^ 2008), five of the 10 photostimulated females were photostimulated again using the same protocol, while the other five photostimulated females and five control birds were also kept indoors but under natural photoperiod. Thus, the aviary experiment consisted of three treatment groups, each containing five birds: (i) natural short photoperiod (ii) one long day and (iii) two long days with an interval of one week in between. One month after the first photostimulation (January 15^th^ 2009) the length of the largest follicle was measured again for each female. One week after the last laparotomy all 15 females were released into the wild at the catching location (Fig. 3).

### Field experiment

We carried out a field experiment to test if laying dates in the wild are restricted by gonadal development of the females. In the Oosterhout (∼150 nest boxes) and Hoge Veluwe study area (∼440 nest boxes) all nest boxes were checked at night for the presence of female great tits (Oosterhout 2009: February 25^th^; 2010: March 1^st^; Hoge Veluwe 2009: March 3^rd^). All females were banded with a uniquely numbered aluminum ring as well as a unique colour band combination. Of the 103 females encountered, we took 69 females into temporary captivity (Oosterhout: 2009 *n* = 20, 2010 *n* = 20, Hoge Veluwe: 2009 *n* = 29; Table 1). Thus, 34 females were not taken into captivity; these control females were put back in the nest box after being ringed and weighed with a pesola spring scale (Table 1). The females taken into captivity were housed indoor in individual cages with *ad libitum* food and water.

Half of the females were kept indoors under a natural light regime (light from 7:30AM to 6:15PM), while the other half were photostimulated (light from 7AM to 3AM; Fig. 3), and thus experienced an increase in day length of 8 hours and 45 minutes. Thus, we created three experimental groups in this field experiment: (i) photostimulated females in captivity (ii) females which experienced natural day length in captivity and (iii) females which experienced natural day length in the field. We chose not to measure gonadal size of the females in the field after release since disturbance in the period just before egg laying could probably affect the timing of egg laying. All females were released at the field site of capture the day after the treatment (day 3, Fig. 3).

Nest boxes at both study sites were checked once a week from the beginning of April onwards to monitor nest building. Once the bottom of the box was covered with nest material, nests were checked daily to determine the exact laying date (date the first egg was laid). During incubation, females were identified by their unique colour code combination by exposing the colour rings with a pen. Laying dates of three females were excluded since they were likely to be replacement clutches (>30 days after the first egg laying dates in that year of that population) of which we had missed the first clutch (removed laying dates are: Oosterhout 2009: April 40^th^; Oosterhout 2010: April 34^th^ and April 61^st^). Results of the analysis did not change after including these data points.

### Statistical analyses

We used a One-sample Wilcoxon signed rank test to test if follicles grew for the photostimulated females in the aviary experiment and a Two-sample Wilcoxon signed rank test to test if gonadal growth was different for females which were photostimulated either once or twice. Statistics presented here were done on the follicle volumes. Results did not change when using follicle length as dependent variable. To test for differences in laying dates in the field experiment we used ANOVAs. Because the experiment was done in Oosterhout in 2009 and 2010 and in the Hoge Veluwe population only in 2009, we divided the analyses of the field experiment in two parts; comparison between study areas in 2009 and comparison between years for the Oosterhout population. All statistics were done using R 2.9.2 [33].
